# Addition of Thermotolerant Nitrifying Bacteria During Pig Manure Composting Enhanced Nitrogen Retention and Modified Microbial Composition

**DOI:** 10.3390/microorganisms13040719

**Published:** 2025-03-23

**Authors:** Biao Liu, Zhaohui Guo, Wei Chen, Zhen Wang, Lijuan Xu, Shuaishuai Gao, Yingben Wu, Yan Zeng, Bingxuan Tang, Minxi Wu, Hongmei Yin

**Affiliations:** Hunan Institute of Microbiology, Hunan Academy of Agricultural Sciences, Changsha 410009, China; liubiao@hnwyy.cn (B.L.);

**Keywords:** pig manure composting, nitrogen loss, microbial community, nitrogen conversion genes

## Abstract

Preventing loss of nitrogen during aerobic manure composting is a critical challenge, and introducing microbial agents with specific functions offers a promising solution. This study aimed to explore how *Bacillus subtilis* F2 (a thermotolerant nitrifying bacterium) affects nitrogen conservation, microbial dynamics, and nitrogen conversion-associated gene abundance during pig manure composting. Relative to the uninoculated controls, adding F2 markedly raised the germination index, nitrate content, and total nitrogen in the final compost, resulting in reduced nitrogen loss. The inoculation led to a distinct succession of bacterial communities, enriching microorganisms associated with fermentation and hydrocarbon degradation, while the fungal communities did not change significantly between the control and treated compost. Furthermore, inoculation markedly increased *amoA* gene levels and decreased *nirK* abundance during the cooling and maturation phases. Significant relationships were detected between nitrogen content, microbial composition, and nitrogen conversion genes in correlation analyses. In summary, the addition of F2 is recommended for bolstering nitrogen retention in the context of composting.

## 1. Introduction

The intensification of animal farming in China has increased the production of manure [1]. While manures are rich sources of nitrogen (N), phosphorus (P), and potassium (K), they also contain harmful substances like pathogens and antibiotics. Aerobic composting provides a practical, eco-friendly strategy for treating livestock manure, transforming it into an effective and safe fertilizer through microbial metabolic activity [2,3]. Organic matter undergoes mineralization and humification during composting, ultimately becoming plant-available nutrients [4]. Nitrogen loss is a significant challenge in composting [5]. Studies have reported that nitrogen loss is mainly due to ammonia (NH_3_) volatilization, accounting for 79–94% of total nitrogen (TN) loss [6,7]. Emission of ammonia not only pollutes the atmosphere but also reduces nitrogen levels in the compost, affecting its nutritional value [8]. Therefore, enhancing nitrogen retention during composting has become a key research focus.

Optimizing composting parameters like the carbon-to-nitrogen (C:N) ratio, moisture, pH, and ventilation can mitigate nitrogen loss, although these must be carefully balanced to avoid adversely impacting this process [9]. It has been found that physicochemical additives, including biochar, zeolite, calcium superphosphate, and phosphogypsum, can significantly limit nitrogen loss. However, these additives are often costly, non-recyclable, and may have adverse effects on soil and crops with the passage of time. In contrast, microbial inoculants offer a cost-effective, eco-friendly alternative for limiting nitrogen loss, accelerating decomposition, and enhancing compost maturity. For instance, Li et al. [10] demonstrated that a composite microbial inoculum extended the thermophilic phase and significantly raised the ammonium (NH_4_^+^-N) and nitrate (NO_3_^−^-N) content in composted pig manure.

The effectiveness of the composting process is predominantly influenced by the temperature profile within the compost pile and the metabolic activity of the microbial community. Compost additives can influence microbial community makeup, thereby affecting nitrogen conversion-related genes and nitrogen metabolism. Ammoxidation is the first and rate-limiting step of nitrification, wherein NH_3_/NH_4_^+^ forms hydroxylamine by ammonia monooxygenase (encoded by *amoA* gene), whereas the reduction process from nitrite (NO_2_^−^-N) to nitric oxide (NO) is mediated through the expression of nitrite reductase (encoded by *nirK* and *nirS*) in denitrifying microorganisms [7]. Guo et al. [11] found that *Bacillus megaterium* and bentonite altered microbial community composition by increasing Firmicutes and Bacteriodetes abundance while reducing Proteobacteria and Actinobacteria abundance, in addition to reducing nitrogen losses by regulating the intensity of nitrification through the ammonia monooxygenase gene (*amoA*) and denitrification through the nitrate reductase genes (*nirK* and *nirS*). These additives thereby reduced ammonia (NH_3_) and nitrous oxide (N_2_O) release. Jiang et al. [12] further found that urease inhibitors markedly enhanced both the richness and diversity of microbial community structures during pig manure composting, thereby increasing *amoA*, *nirK*, and *nirS* abundance. Significantly higher TN and NO_3_^−^-N levels were observed in the final compost product following such urease inhibitor treatment relative to control compost. While research has extensively examined bacterial community dynamics, studies on fungal communities remain limited. Fungi contribute to the mineralization of organic material, particularly under extreme conditions such as high temperatures and pH [13,14]. Li et al. [15] observed the dominant fungi to include Ascomycota, Basidiomycota, and Mucoromycota during the processing of pig manure, with a 10% addition of pine leaf biochar enhancing fungal diversity, composting efficiency, and compost quality.

Nitrification is critical for converting NH_4_^+^-N into NO_2_^−^-N and NO_3_^−^-N, thereby reducing NH_3_ emissions [7], and the introduction of exogenous nitrifying microbes is thus a promising means of enhancing nitrification to lower nitrogen loss and enhance compost quality. Naghdi et al. [16] demonstrated that inoculating vegetable waste compost with nitrifying bacteria accelerated ammonium conversion to nitrate. Similarly, Zhao et al. [17] reported that a thermotolerant nitrifying bacterial inoculum decreased NH_3_ and N_2_O emissions in sludge composts while increasing the nitrogen content. However, most nitrifying bacteria are intolerant to temperatures exceeding 40 °C [7,18], limiting their effectiveness when temperatures increase during composting. NH_4_^+^-N accumulation and NH_3_ release primarily occur during this high-temperature composting phase. At present, thermotolerant nitrifying microbes that can help prevent these nitrogen losses are lacking, and there is a need to further evaluate the effects of nitrifying microbial strains on nitrogen content given that exogenous inoculation may markedly alter any endogenous nitrifying and denitrifying microbes present in the composting environment.

To address this limitation, this study inoculated pig manure compost with a thermotolerant nitrifying bacterial strain, *Bacillus subtilis* F2. The objectives were to: (1) analyze the impact of microbial addition on compost maturity and available nitrogen levels and (2) investigate shifts in microbial community compositions and the levels of nitrogen conversion-related genes during the composting process.

## 2. Materials and Methods

### 2.1. Bacterial Preparation and Composting Materials

*Bacillus subtilis* F2, isolated by the authors’ research group, demonstrates robust nitrification capabilities and high-temperature tolerance. The strain was cultured in LB medium and fermented at 55 °C for 72 h. The bacterial suspension was then centrifuged, and the sediment was resuspended in sterile water at ~2.0 × 10^9^ CFU/mL for inoculation purposes.

The fresh pig manure and sawdust were obtained from Chenhe Ecological Agriculture Co., Ltd. (Shaoyang, China). Table 1 shows the physicochemical properties of the composting materials. The experiment was carried out at Chenhe Ecological Agriculture Co., Ltd. from September to October, 2023. To produce a C:N ratio of 25:1, pig manure and sawdust were blended in a 9:1 (*w*/*w*) ratio, with denionized water added to yield a moisture content of 60%. Two composting treatments were established with identical pile dimensions (2.0 m × 2.0 m × 1.5 m). For the experimental group (T), 1% *Bacillus subtilis* F2 (relative to wet compost weight) was added, while the control group (CK) received no inoculation. To maintain adequate aeration, the compost piles were turned on days 0, 3, 7, 10, 14, 21, 28, and 40. Samples were collected on the same days and divided into two portions. One was freeze-dried and kept at −80 °C for sequencing and analysis of nitrogen-converting genes, while the other was air-dried for determining the NH_4_^+^-N, NO_3_^−^-N, TN, and germination index (GI).

### 2.2. Analysis of Physical and Chemical Properties

A digital thermometer (Hengshui Zhengxu Electronic Technology Co. Ltd., Hengshui, China) was used daily at 9:00 AM to measure compost temperatures, while the ambient air temperature was determined based on prevailing environmental conditions. NH_4_^+^-N and NO_3_^−^-N concentrations were assessed utilizing the distilled sulfuric acid titration method and ultraviolet spectrophotometry, respectively, in accordance with the Chinese Agricultural Industry Standard (NY/T 1116-2014) [19]. TN contents were assessed using the Kjeldahl method, following the Chinese standards for organic fertilizers (NY/T 525-2021) [20]. Compost maturity was assessed using cucumber seed germination, and the germination index (GI) was determined as:

GI = (Germination rate of seed × Root length of compost extract solution treatment)/(Germination rate of seeds × Root length of distilled water control)

### 2.3. High-Throughput 16S rDNA and ITS Sequencing

Following thermal analyses during the composting process, freeze-dried samples were chosen from days 14 (thermophilic phase), 28 (cooling phase), and 40 (maturation phase) for high-throughput sequencing of the 16S rDNA genes and ITS regions by Shanghai Majorbio Biotechnology Co. Ltd. (Shanghai, China). To extract DNA, an E.Z.N.A.^®^ soil DNA kit (Omega Bio-tek, Norcross, GA, USA) was utilized as directed, followed by 1% agarose gel electrophoresis and the NanoDrop2000 assessment of RNA quality. The 338F (5′-ACTCCTACGGGAGGCAGCAG-3′) and 806R (5′-GGACTACHVGGGTWTCTAAT-3′) primers were utilized for amplification of the V3-V4 regions of the bacterial 16S rDNA genes, whereas the ITS1F (5′-CTTGGTCATTTAGAGGAAGTAA-3′) and ITS2R (5′-GCTGCGTTCTTCATCGATGC-3′) primers enabled fungal ITS1 region amplification. PCR product quality was evaluated using 2% agarose gel electrophoresis and purified with the AxyPrep DNA Gel Extraction Kit (Axygen Biosciences, Union City, CA, USA). The products were evaluated using 2% agarose gel electrophoresis and a Quantus™ Fluorometer (Promega, San Luis Obispo, CA, USA). Libraries were prepared with the NEXTFLEX Rapid DNA-Seq Kit (Bioo Scientific Corporation, Austin, TX, USA)and sequenced on an Illumina MiSeq PE300 platform (Illumina, San Diego, CA, USA).

Raw sequences were processed with Fastp (version 0.19.6) for quality control and Flash software (version 1.2.11) for assembly. Comprehensive sequence analysis, including taxonomic annotation, differential analysis, functional prediction, and correlations between environmental factors and species, was performed on the Majorbio Cloud Platform.

### 2.4. Quantitative Analysis of Nitrogen Conversion-Related Genes

Nitrogen conversion-related gene (*amoA*, *nirK*, and *nirS*) levels were measured by real-time qPCR using the primers shown in Table 2, as described by Liu et al. [21]. An ABI 7300 real-time PCR system was utilized. Each 10 μL reaction mixture included 5 μL of 2× ChamQ SYBR Color qPCR Master Mix, 0.4 μL of individual primers, 0.2 μL of 50× ROX Reference Dye 1, 1 μL of template DNA, and 3 μL of ddH_2_O.PCR settings included 95 °C for 3 min, followed by 40 cycles of 95 °C for 5 s, 58 °C for 30 s, and 72 °C for 60 s. Gene copy numbers were quantified using standard curves.

### 2.5. Data Analysis

All results were given as means and standard deviations, derived from triplicate experiments. Statistical analysis of physical-chemical properties and nitrogen conversion-related gene abundances was performed using SPSS 25.0. The compost samples from CK and T collected on the same day were subjected to a Student’s *t*-test for analysis, whereas the variations between CK and T throughout the composting process were assessed using a one-way ANOVA for multiple comparisons. Graphs were created using Microsoft Excel 2010. Raw bacterial and fungal sequences were deposited in the NCBI Sequence Read Archive (SRA) database under accession numbers PRJNA1094671 and PRJNA1095178, respectively. 

## 3. Results and Discussion

### 3.1. Composting-Related Temperature, GI, NH_4_^+^-N, NO_3_^–^-N, and TN Changes

**Figure 1:**
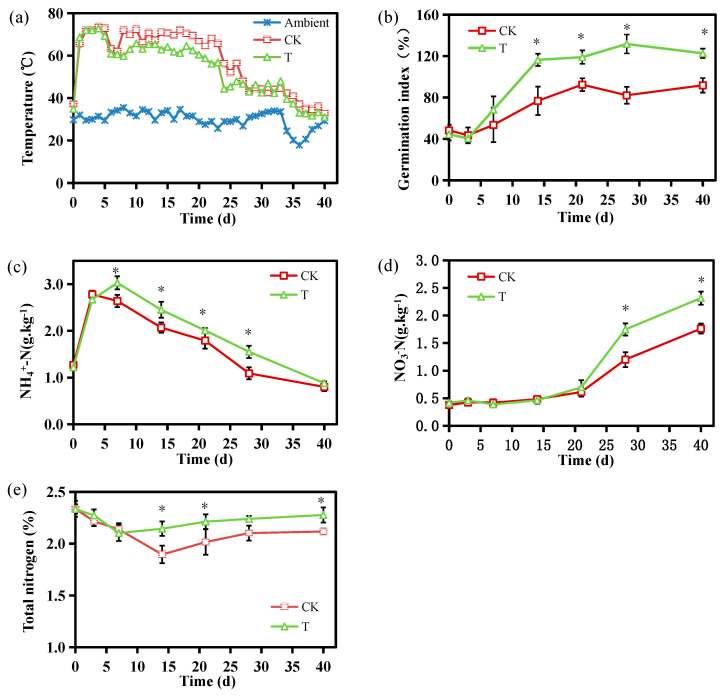
Temperature (**a**), GI (**b**), NH_4_^+^-N (**c**), NO_3_^−^-N (**d**), and TN (**e**) profiles during the composting process (CK, no additives; T, 1% *Bacillus subtilis* F2). * *p* < 0.05, Student’s *t*-test; CK vs. T.

Temperature is crucial for evaluating composting effectiveness, as it reflects the sanitation of the compost and microbial community activity within the pile [22]. The temperature profiles of both the control (CK) and treatment (T) groups followed similar patterns, with a rapid rise into the thermophilic phase (>50 °C) on day 1, followed by cooling and maturation phases (Figure 1a). Peak temperatures were reached on day 4, with CK reaching 73.5 °C and T slightly lower at 72.8 °C. The thermophilic phase lasted 26 days in CK but was shortened by 3 days in T. According to the Chinese National Standard GB/T 36195-2018 [23], these durations meet hygienic requirements by effectively eliminating most pathogens, weed seeds, and insect eggs. As easily accessible organic matter diminished, the temperature of all treatments gradually declined, eventually reaching ambient levels by the end of the composting process. Interestingly, while microbial agents are often reported to extend the thermophilic stage [17], the addition of *Bacillus subtilis* F2 shortened this phase, indicating its distinct influence on composting dynamics. The elevated temperatures in compost can accelerate NH_4_^+^-N conversion into volatile ammonia gas [7]. The temperature-regulating effect of B. subtilis F2 likely contributed to improved NH_4_^+^-N retention during composting.

The GI is a crucial indicator of compost maturation [3]. Both treatments experienced their lowest GI values on day 3, at 43.50% for CK and 40.57% for T (Figure 1b). Similar observations have previously been described, attributing the decline to short-chain volatile fatty acid and ammonia production in the early stages [24]. As composting advanced, the GI steadily rose owing to the decomposition of phytotoxic substances. Compost is considered mature and free from phytotoxins when the GI exceeds 80% [25]. While CK surpassed this threshold on day 21, T reached it earlier, on day 14, demonstrating that *B. subtilis* F2 accelerated the composting process. The final GI values were 91.83% for CK and 122.66% for T, highlighting a significant improvement in compost maturity with microbial inoculation.

The NH_4_^+^-N concentration followed a similar trend in both groups, showing a sharp increase during the initial three days (Figure 1c). CK reached its peak NH4+-N concentration of 2.78 g/kg on day 3, while T peaked at 2.64 g/kg on day 7. This pattern can be ascribed to the rapid mineralization of organic nitrogen through ammonification [26]. NH_4_^+^-N concentrations then gradually declined due to ammonia volatilization and NH_4_^+^-N conversion to other nitrogenous forms via nitrification and denitrification [27]. Between days 7 and 28, NH_4_^+^-N levels were consistently lower in CK than in T, likely as a result of higher temperatures in CK causing greater ammonia loss. In the final compost, NH_4_^+^-N concentrations were comparable in both groups, suggesting that inoculation with F2 facilitated NH_4_^+^-N nitrification later during composting.

During the thermophilic phase, NO_3_^−^-N concentrations were relatively low (Figure 1d), probably because high temperatures inhibit nitrifying bacteria activity [28]. As temperatures declined, the growth and activities of the nitrifying bacteria intensified, accompanied by a rise in the NO_3_^−^-N content, which peaked when composting was complete. Relative to CK, the final NO_3_^−^-N concentration in T was higher, at 2.317 g/kg, indicating that *B. subtilis* F2 inoculation enhanced nitrate accumulation.

TN levels in both CK and T decreased during the initial phase, reaching their lowest values on days 14 and 7, respectively (Figure 1e). This decline is due to the rapid biodegradation of organic nitrogen and significant NH_3_ volatilization [29]. TN levels then gradually increased in both groups due to the degradation of organic matter and fixation of nitrogen. On completion of composting, the TN contents in T surpassed those in CK. TN loss was also lower in T (28.7%) compared to CK (37.9%) (Table S1), demonstrating that inoculation with F2 effectively reduced nitrogen loss.

### 3.2. Microbial Community Dynamics over the Course of Composting

#### 3.2.1. Bacterial Community and Functional Dynamics

**Figure 2:**
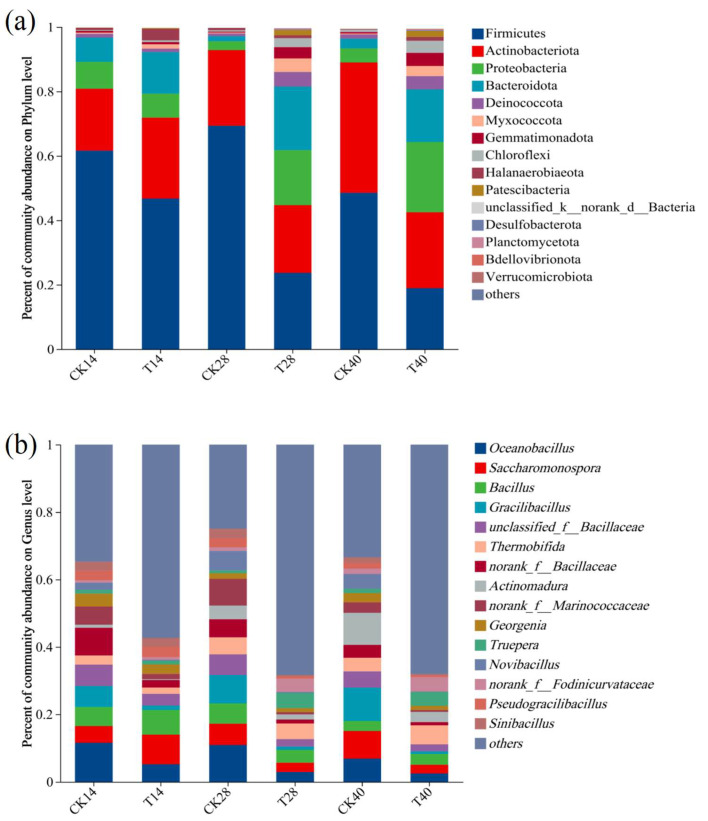
Alterations in bacterial communities during the composting process. (**a**) Phyla; (**b**) Genera. CK14, CK28, and CK40 correspond to non-inoculated samples collected on days 14, 28, and 40, respectively, whereas T14, T28, and T40 respectively correspond to samples inoculated with 1% *Bacillus subtilis* F2 and collected on these same respective days.

The phyla exhibiting the greatest abundance during the composting process were Firmicutes, Actinobacteria, Proteobacteria, and Bacteroidota, with their combined relative abundance exceeding 80% across all stages (Figure 2a). During the thermophilic and cooling phases, Firmicutes were most abundant in both groups, reflecting their ability to produce heat-tolerant spores [30]. Relative to CK, the T group had a lower relative abundance of Firmicutes on days 14, 28, and 40. This contrasts with previous studies where Bacillus-based inoculants increased Firmicutes abundance [10,11]. Conversely, the T group displayed higher relative levels of Proteobacteria, Bacteroidota, Myxococcota, Gemmatimonadota, and Chloroflexi during cooling and maturation phases (Figure S1). Proteobacteria and Bacteroidota are known to be involved in nitrogen conversion [31], suggesting that *B. subtilis* F2 inoculation influenced nitrogen metabolism by reshaping the microbial communities.

The most abundant genera were *Oceanobacillus*, *Saccharomonospora*, *Bacillus*, *Gracilibacillus*, *unclassified_f__Bacillaceae*, *Thermobifida*, *norank_f__Bacillaceae*, *Actinomadura*, *norank_f__Marinococcaceae*, and *Georgenia* (Figure 2b). The total relative abundance of these bacteria was significantly lower in T in comparison with CK. Throughout the composting process, CK exhibited higher relative abundances of *Oceanobacillus*, *Gracilibacillus*, *norank_f__Bacillaceae*, *Actinomadura*, *norank_f_Marinococcaceae*, and *Georgenia*. On day 14, the T group exhibited greater relative abundance of *Saccharomonospora* and *Bacillus* than CK, but these trends diverged on day 28.

**Figure 3:**
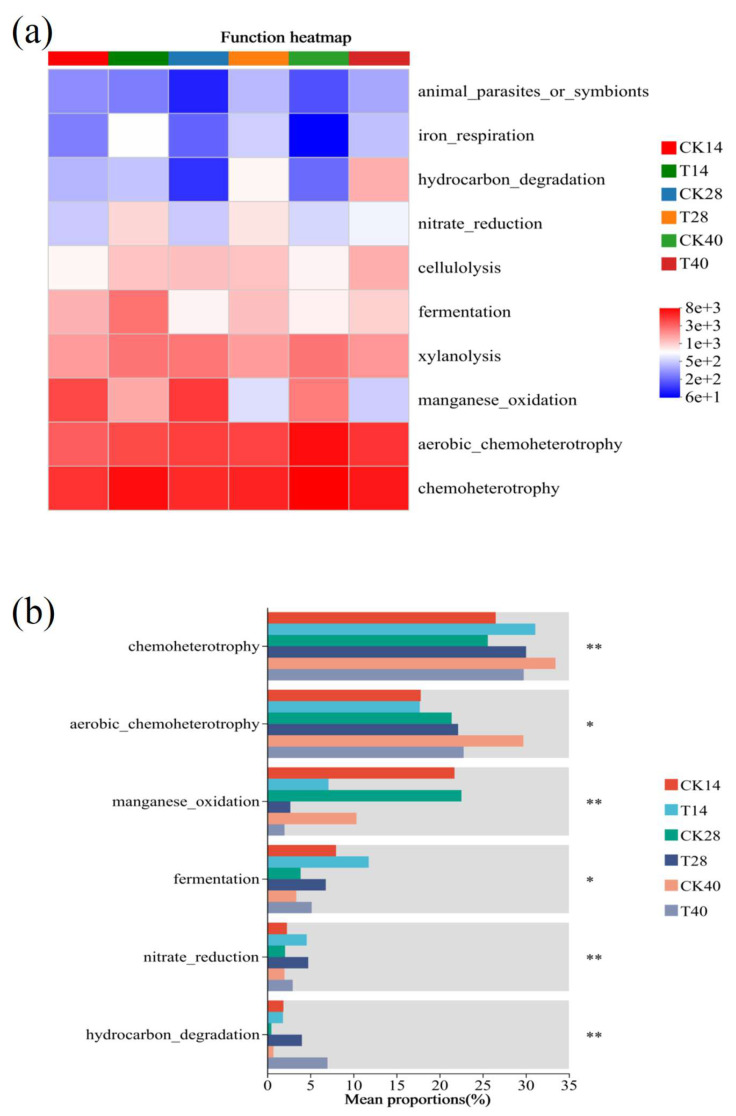
Bacterial functional succession. (**a**) Bacterial function heatmap. (**b**) Analyses of differences in bacterial function. One-way ANOVA, * *p* < 0.05, ** *p* < 0.01.

The FAPROTAX database was utilized for functional predictions of the bacterial communities in CK and T. The relative abundances of bacterial functions during different composting phases are illustrated in Figure 3a. The bacterial communities in the composts exhibited high diversity and abundance in ecological functions, with dominant roles including chemoheterotrophy (specifically aerobic chemoheterotrophy), manganese oxidation, xylanolysis, fermentation, cellulolysis, nitrate reduction, and hydrocarbon degradation. Among these, chemoheterotrophy was the most prevalent function throughout the composting process, playing a pivotal role in decomposing organic waste materials and releasing essential nutrients and energy. On days 14 and 28, the proportion of chemoheterotrophy was greater in T compared to CK. This suggests that the decomposition reaction of organic matter is more intense in the T treatment. Manganese oxidation was also significantly represented, potentially serving as a nutrient source for bacteria while enhancing their resilience to adverse environmental conditions. Bacteria with manganese-oxidizing capabilities are primarily associated with the phyla Firmicutes, Actinobacteria, and Proteobacteria [32]. Since the F2 inoculant significantly influenced the relative abundances of these phyla, notable differences in manganese-oxidizing bacterial populations were observed between the CK and T groups. The function related to nitrate reduction was more abundant in T, possibly attributed to the higher concentration in compost samples. Composting involves complex fermentation and organic matter degradation, as demonstrated in Figure 3b, adding F2 markedly elevated functions associated with hydrocarbon degradation and fermentation, thereby accelerating compost maturity.

#### 3.2.2. Fungal Community Succession

**Figure 4:**
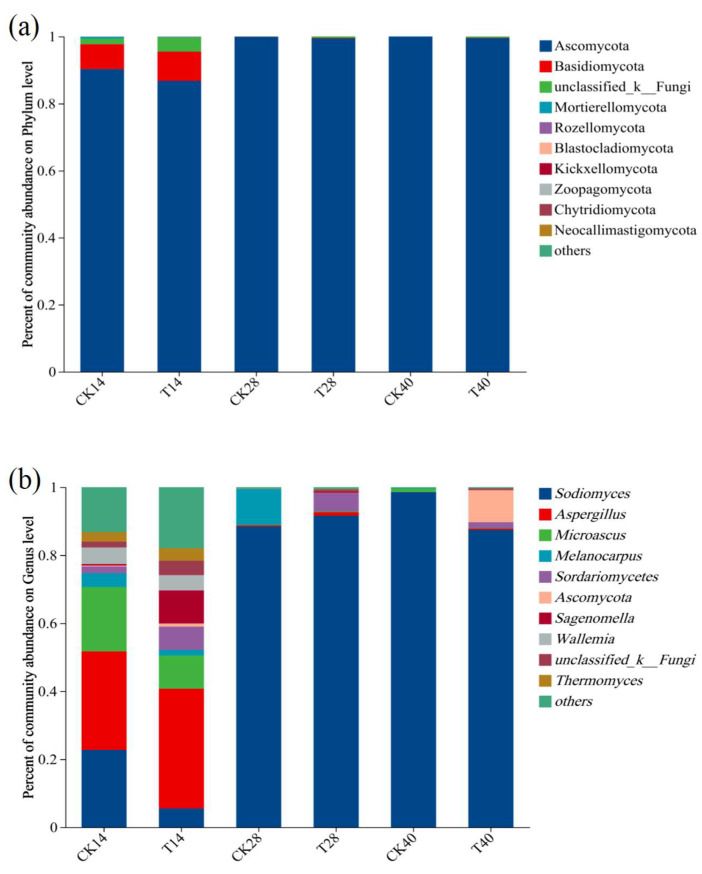
Variation in the fungal community: (**a**) Phyla; (**b**) Genera.

The relative levels of fungal phyla and genera are presented in Figure 4a and Figure 4b, respectively. The phylum Ascomycota predominated in the compost, accounting for over 86% of fungal communities during the thermophilic stage. During the phases of cooling and maturation, its relative abundance increased to over 99%. Ascomycota plays a critical role in lignocellulose degradation due to its production of spores capable of withstanding high temperatures during composting [33]. Basidiomycota was the second most common fungal phylum, representing 0% to 8.70% of the total fungi. Similar findings, highlighting the dominance of Ascomycota and Basidiomycota, have been previously described [15,34]. The incorporation of F2 did not significantly alter fungal phylum succession.

Notable differences in fungal genera were observed compared with earlier investigations [15,27]. During the thermophilic phase, dominant fungal genera included *Sodiomyces*, *Aspergillus*, *Microascus*, *Melanocarpus*, *unclassified_c__Sordariomycetes*, *Sagenomella*, and *Wallemia*. As composting progressed, the relative abundance of *Sodiomyces* increased dramatically in both groups, comprising 87.4–98.5% during the cooling and maturation phases. *Sodiomyces* is associated with protein degradation, as reported by Grum-Grzhimaylo et al. [35], suggesting that protein degradation primarily occurs later during composting. The analysis of dominant fungal genera revealed similar patterns to those observed at the phylum level, with no marked difference between CK and T.

### 3.3. Effects of Bacterial Addition on the Levels of Nitrogen-Converting Genes

**Figure 5:**
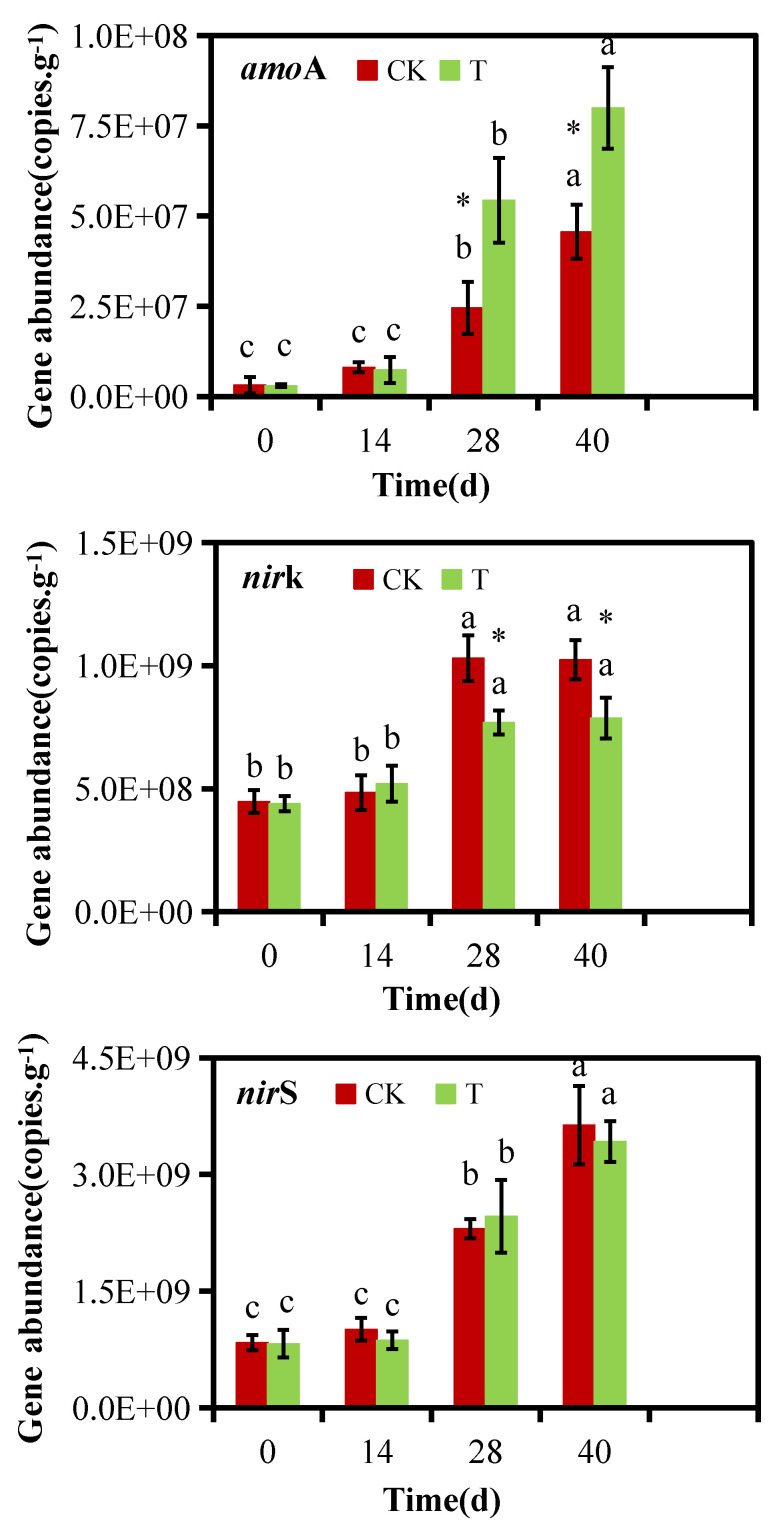
Changes in nitrogen conversion-related gene abundance. The error bars represent the standard deviation of the data. * indicates significant differences between treatments in the same time (*p* < 0.05). Different lowercase letters indicate significant differences among treatments (*p* < 0.05).

The abundances of key nitrogen-conversion genes associated with microbial nitrification (*amoA*) and denitrification (*nirS* and *nirK*) are illustrated in Figure 5. The *amoA* gene facilitates the conversion of ammonia to nitrite, directly impacting NH_4_^+^-N levels and influencing NH_3_ emissions. During the first 14 days, the levels of *amoA* remained low, likely because high temperatures inhibit the growth of ammonia-oxidizing microorganisms [36]. As temperatures decreased, these microbial communities recovered, leading to increased *amoA* gene copy numbers. This trend aligns with earlier results [11,12]. While the two groups were comparable during the initial and thermophilic stages, the introduction of F2 significantly raised the levels of *amoA* during the cooling and maturation stages. These findings suggest that F2 inoculation enhanced the oxidation of NH_4_^+^-N by *amoA*, thus limiting NH_3_ emissions during composting.

The levels of denitrification genes (*nirS* and *nirK*) exceeded those of the nitrification gene (*amoA*), indicating more active denitrification than nitrification during composting. This agrees with earlier results [12]. Among the denitrification genes, *nirS* showed higher abundance than *nirK*, a result also observed by Xiong et al. [37]. However, contrasting outcomes have been reported, with *nirK* being more abundant in composts of agricultural waste [11,38]. This may have been due to variations in the species and abundances of denitrifying microbes across compost materials. During cooling and maturation, the copy numbers of both *nirS* and *nirK* increased significantly compared to the thermophilic phase, reflecting heightened denitrification activity at lower temperatures. While the *nirS* gene was comparable in the CK and T groups, the *nirK* gene copy number was significantly lower in the T group during these phases. A lower abundance of the *nirK* gene may reduce nitrite reduction, leading to decreased NO emissions and nitrogen loss during composting.

### 3.4. Associations Between Microbes, Available Nitrogen, and Nitrogen Conversion-Associated Genes

**Figure 6:**
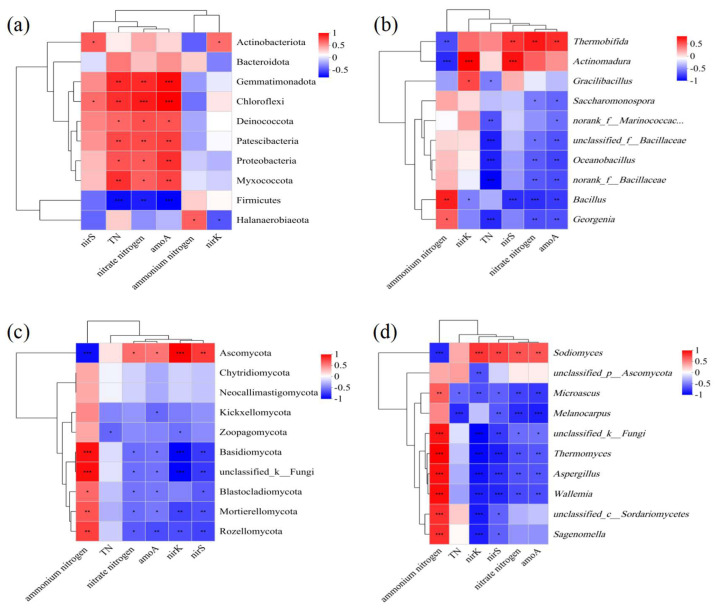
Heatmaps of Spearman correlation coefficients between microbial communities, nitrogen types, and genes associated with nitrogen conversion. Positive and negative correlations are respectively shown in red and blue. * *p* < 0.05, ** *p* < 0.01, *** *p* < 0.001.

Strong positive correlations were evident between TN, NO_3_^−^-N, and *amoA* with the bacterial phyla Gemmatimonadota, Chloroflexi, Deinococcota, Patescibacteria, Proteobacteria, and Myxococcota. Conversely, these variables showed negative correlations with Firmicutes (Figure 6a). In terms of genera, TN, NO_3_^−^-N, and *amoA* were negatively associated with *Oceanobacillus*, *unclassified_f_Bacillaceae*, *norank_f_Bacillaceae*, and *Georgenia* (Figure 6b). Previous research has identified many of these bacteria, such as Proteobacteria, as key players in nitrogen conversion processes, including nitrogen fixation [39]. The changes in bacterial communities (Figure S1) suggest that F2 inoculation increased TN content, nitrate concentration, and *amoA* gene abundance by modulating the relative abundance of these bacteria. The association between TN content and specific bacterial phyla or genera was consistent with the correlation observed between nitrate concentration and *amoA* gene abundance within the same bacterial groups. This indicates that F2 inoculation altered the composition of bacterial communities, enhancing *amoA* gene abundance and promoting nitrogen retention during composting.

Spearman correlation analyses were subsequently conducted using R software (version 3.3.1) with the pheatmap package to explore the relationships between nitrogen levels, nitrogen conversion-associated genes, and the dominant fungal phyla or genera (Figure 6c,d). The dominant phylum during composting, Ascomycota, was positively associated with NO_3_^−^-N, *amoA*, *nirS*, and *nirK*, whereas it was negatively linked with NH_4_^+^-N. Basidiomycota, conversely, was markedly associated with all of these indices. The genus *Sodiomyces* was positively linked with NO_3_^−^-N, *amoA*, *nirS*, and *nirK*, whereas it was negatively associated with NH_4_^+^-N. During cooling and maturation, the relative *Sodiomyces* abundance rose significantly relative to that during the thermophilic phase (Figure 4b), indicating that nitrification and denitrification activity levels were enhanced during the former two of these phases relative to the thermophilic phase. In past reports, nitrification and denitrification have been shown to primarily take place during the cooler composting stages [11,40]. In contrast, *Aspergillus*, *Miroascus*, *Thermomyces*, *Wallemia*, and *unclassified_k_Fungi* were negatively associated with NO_3_^−^-N, *amoA*, *nirS*, and *nirK*, although they were positively linked with NH_4_^+^-N. Most dominant fungi exhibited no significant correlations with TN levels (Figure 6), providing further confirmation that F2 inoculation primarily increased total nitrogen levels in compost through changes in the structures of bacterial communities.

## 4. Conclusions

(1) These results offer clear evidence that *B. subtilis* F2 addition was sufficient to promote the improved maturity of the final product of pig manure composting. Bacterial inoculation led to increased nitrate by 31.4% and TN concentrations by 7.6% in the final compost while also limiting nitrogen loss relative to that in the CK group.

(2) Microbial analyses revealed no clear changes in fungal community composition when comparing the CK and T groups, whereas inoculation markedly altered the bacterial community and affected nitrogen conversion-related gene abundance. The F2 inoculation demonstrated significantly elevated relative abundances of Proteobacteria, Bacteroidota, Myxococcota, Gemmatimonadota, and Chloroflexi throughout both the cooling and maturation phases. Notably, the copy number of the crucial nitrification gene, *amoA*, showed substantial increases of 122% and 75% in the inoculated compost samples during the cooling and maturation phases, respectively. In contrast, the denitrification gene *nirK* displayed divergent patterns between the control and treatment groups. In correlation analyses, these changes were confirmed to be closely linked to nitrogen conversion and the abrogation of TN loss during pig manure composting.

## Figures and Tables

**Table 1:** Physicochemical characteristics of the raw materials.

	Moisture (%)	pH	Total Carbon (%) ^a^	Total Nitrogen (%) ^a^	C/N Ratio ^a^
Pig manure	58.82 ± 1.32	8.13 ± 0.04	35.43 ± 0.52	2.05 ± 0.10	17.33 ± 0.61
Sawdust	8.28 ± 0.34	6.66 ± 0.05	52.12 ± 0.03	0.78 ± 0.02	67.11 ± 0.32

Data are means of three replicates ± standard deviation. ^a^ Measurement based on dry weight.

**Table 2:** Primer sequences.

Gene	Primer	Primer Sequence (5′–3′)
*amoA*	bamoA1F	GGGGTTTCTACTGGTGGT
	bamoA2R	CCCCTCKGSAAAGCCTTCTTC
*nirS*	Cd3aF	GTSAACGTSAAGGARACSGG
	R3cdR	GASTTCGGRTGSGTCTTGA
*nirK*	nirK1aCuF	ATCATGGTSCTGCCGCG
	nirKR3CuR	GCCTCGATCAGRTTGTGGTT

## Data Availability

The original contributions presented in this study are included in the article/Supplementary Materials. Further inquiries can be directed to the corresponding author.

## Supplementary Materials

The following supporting information can be downloaded at: www.mdpi.com/article/10.3390/microorganisms13040719/s1, Figure S1: Significant differences in bacterial community composition at the (a) phylum and (b) genera levels when comparing the CK and T groups during composting; Table S1: Total nitrogen loss.

## References

[1] Yao X., Zhou H., Meng H., Ding J., Shen Y., Cheng H., Zhang X., Li R., Fan S. (2021). Amino acid profile characterization during the co-composting of a livestock manure and maize straw mixture. J. Clean. Prod..

[2] Chen W., Liao X., Wu Y., Liang J., Mi J., Huang J., Zhang H., Wu Y., Qiao Z., Li X. (2017). Effects of different types of biochar on methane and ammonia mitigation during layer manure composting. Waste Manag..

[3] Ji Z., Zhang L., Liu Y., Li X., Li Z. (2023). Evaluation of composting parameters, technologies and maturity indexes for aerobic manure composting: A meta-analysis. Sci. Total Environ..

[4] Onwosi C.O., Igbokwe V.C., Odimba J.N., Eke I.E., Nwankwoala M.O., Iroh I.N., Ezeogu L.I. (2017). Composting technology in waste stabilization: On the methods, challenges and future prospects. J. Environ. Manag..

[5] Ren X., Wang Q., Li R., Chang C.C., Pan J., Zhang Z. (2020). Effect of clay on greenhouse gas emissions and humification during pig manure composting as supported by spectroscopic evidence. Sci. Total Environ..

[6] Beck-Friis B., Smårs S., Jonsson H., Kirchmann H. (2001). SE—Structures and environment: Gaseous emissions of carbon dioxide, ammonia and nitrous oxide from organic household waste in a compost reactor under different temperature regimes. J. Agric. Eng. Res..

[7] Shan G., Li W., Gao Y., Tan W., Xi B. (2021). Additives for reducing nitrogen loss during composting: A review. J. Clean. Prod..

[8] Yang Y., Awasthi M.K., Ren X., Guo H., Lv J. (2019). Effect of bean dregs on nitrogen transformation and bacterial dynamics during pig manure composting. Bioresour. Technol..

[9] Zhang Z., Liu D., Qiao Y., Li S., Chen Y., Hu C. (2021). Mitigation of carbon and nitrogen losses during pig manure composting: A meta-analysis. Sci. Total Environ..

[10] Li C., Li H., Yao T., Su M., Li J., Liu Z., Xin Y., Wang L., Chen J., Gun S. (2020). Effects of microbial inoculation on enzyme activity, available nitrogen content, and bacterial succession during pig manure composting. Bioresour. Technol..

[11] Guo H., Gu J., Wang X., Nasir M., Yu J., Lei L., Wang J., Zhao W., Dai X. (2020). Beneficial effects of bacterial agent/bentonite on nitrogen transformation and microbial community dynamics during aerobic composting of pig manure. Bioresour. Technol..

[12] Jiang J., Wang Y., Guo F., Zhang X., Dong W., Zhang X., Zhang X., Zhang C., Cheng K., Li Y. (2020). Composting pig manure and sawdust with urease inhibitor: Succession of nitrogen functional genes and bacterial community. Environ. Sci. Pollut. R..

[13] Wang K., Yin X.B., Mao H.L., Chu C., Tian Y. (2018). Changes in structure and function of fungal community in cow manure composting. Bioresour. Technol..

[14] Duan Y.M., Awasthi S.K., Chen H.Y., Liu T., Zhang Z.Q., Zhang L.S., Awasthi M.K., Taherzadeh M.J. (2019). Evaluating the impact of bamboo biochar on the fungal community succession during chicken manure composting. Bioresour. Technol..

[15] Li J., Bao H.Y., Xing W.J., Yang J., Liu R.F., Wang X., Lv L.H., Tong X.G., Wu F.Y. (2020). Succession of fungal dynamics and their influence on physicochemical parameters during pig manure composting employing with pine leaf biochar. Bioresour. Technol..

[16] Naghdi M., Cledon M., Brar S., Ramirez A. (2018). Nitrification of vegetable wastes using nitrifying bacteria. Ecol. Eng..

[17] Zhao Y., Li W.G., Chen L., Meng L.Q., Zhang S.M. (2023). Impacts of adding thermotolerant bacteria on nitrogenous gas emissions and bacterial community structure during sewage sludge composting. Bioresour. Technol..

[18] Chen M., Chen Y., Dong S., Lan S., Zhou H., Tan Z., Li X. (2018). Mixed nitrifying bacteria culture under different temperature dropping strategies: Nitrification performance, activity, and community. Chemosphere.

[19] (2014). Fertilizers—Determination of Nitrate Nitrogen, Ammonium Nitrogen, Amide Nitrogen Contents.

[20] (2021). Organic Fertilizer.

[21] Liu B., Chen W., Wang Z., Guo Z., Li Y., Xu L., Wu M., Yin H. (2024). The impact of *Bacillus coagulans* X3 on available nitrogen content, bacterial community composition, and nitrogen functional gene levels when composting cattle manure. Agronomy.

[22] Manu M.K., Kumar R., Garg A. (2017). Performance assessment of improved composting system for food waste with varying aeration and use of microbial inoculum. Bioresour. Technol..

[23] (2018). Technical Specification for Sanitation Treatment of Livestock and Poultry Manure.

[24] Zhao Y., Li W., Chen L., Meng L., Zheng Z. (2020). Effect of enriched thermotolerant nitrifying bacteria inoculation on reducing nitrogen loss during sewage sludge composting. Bioresour. Technol..

[25] Zeng G., Huang D., Huang G., Hu T., Jiang X., Feng C., Chen Y., Tang L., Liu H. (2007). Composting of lead-contaminated solid waste with inocula of white-rot fungus. Bioresour. Technol..

[26] Li Y., Li W., Liu B., Wang K., Su C., Wu C. (2013). Ammonia emission and biodegradation of organic carbon during sewage sludge composting with different extra carbon source. Int. Biodeterior. Biodegrad..

[27] Tian X., Gao R., Li Y., Liu Y., Zhang X., Pan J., Tang K., Scriber K.E., Amoah I.D., Zhang Z. (2023). Enhancing nitrogen conversion and microbial dynamics in swine manure composting process through inoculation with a microbial consortium. J. Clean. Prod..

[28] Jiang J., Liu X., Huang Y., Huang H. (2015). Inoculation with nitrogen turnover bacterial agent appropriately increasing nitrogen and promoting maturity in pig manure composting. Waste Manag..

[29] Awasthi M.K., Duan Y., Awasthi S.K., Liu T. (2020). Effect of biochar and bacteria inoculum addition on cow dung composting. Bioresour. Technol..

[30] Xue S.D., Zhou L.N., Zhong M.Z., Awasthi A.K., Mao H. (2021). Bacterial agents affected bacterial community structure to mitigate greenhouse gas emissions during sewage sludge composting. Bioresour. Technol..

[31] Zhu L., Huang C.H., Li W., Wu W.X., Tang Z.R., Tian Y., Xi B.D. (2023). Ammonia assimilation is key for the preservation of nitrogen during industrial-scale composting of chicken manure. Waste Manag..

[32] Martínez-Ruiz B.E., Cooper M., Fastner J., Szewzyk U. (2020). Manganese-oxidizing bacteria isolated from natural and technical systems remove cylindrospermopsin. Chemosphere.

[33] Wu X.Y., Amanze C., Yu Z.J., Li J.K., Liu Y.D., Shen L., Yu R.L., Wu X.L., Xu X.W., Tan S.Y. (2022). Evaluation of fungal community assembly and function during food waste composting with *Aneurinibacillus* sp. LD3 inoculant. Bioresour. Technol..

[34] Neher D.A., Weicht T.R., Bates S.T., Leff J.W., Fierer N. (2013). Changes in bacterial and fungal communities across compost recipes, preparation methods, and composting times. PLoS ONE.

[35] Grum-Grzhimaylo A.A., Falkoski D.L., Heuvel J.V.D., Valero-Jimenez C.A., Min B., Choi I.G., Lipzen A., Daum C.G., Aanen D.K., Tsang A. (2018). The obligate alkalophilic soda-lake fungus *Sodiomyces alkalinus* has shifted to a protein diet. Mol. Ecol..

[36] Reed S., Knez M., Uzan A., Stangoulis J.C.R., Glahn R.P., Koren O., Tako E. (2018). Alterations in the gut (*Gallus gallus*) microbiota following the consumption of zinc biofortified wheat (*Triticum aestivum*)-based diet. J. Agric. Food Chem..

[37] Xiong J.P., Ma S.S., He X.Q., Han L.J., Huang G.Q. (2021). Nitrogen transformation and dynamic changes in related functional genes during functional-membrane covered aerobic composting. Bioresour. Technol..

[38] Zhang L., Zeng G., Zhang J., Chen Y., Yu M., Lu L., Li H., Zhu Y., Yuan Y., Huang A. (2015). Response of denitrifying genes coding for nitrite (*nirK* or *nirS*) and nitrous oxide (*nosZ*) reductases to different physico-chemical parameters during agricultural waste composting. Appl. Microbiol. Biotechnol..

[39] Tom O.D., Christopher Q., Alon S., Özcan C.E., Tm L.S., Michael S.R., Sandra L.M., Sebastian L., Murat E.A. (2018). Nitrogen-fixing populations of Planctomycetes and Proteobacteria are abundant in surface ocean metagenomes. Nat. Microbiol..

[40] Mironov V., Vanteeva A., Sokolova D., Merkel A., Nikolaev Y. (2021). Microbiota dynamics of mechanically separated organic fraction of municipal solid waste during composting. Microorganisms.

