# Supplementary material for: Addition of Thermotolerant Nitrifying Bacteria During Pig Manure Composting Enhanced Nitrogen Retention and Modified Microbial Composition
Source: Microorganisms. 2025 Mar 23;13(4):719. doi: 10.3390/microorganisms13040719 (PMC12029871; doi:10.3390/microorganisms13040719)
Supplement: Supplementary file 1 [file microorganisms-13-00719-s001.zip › microorganisms-3501383-supplementary.pdf]

Table S1. Total nitrogen loss.

Treatment	Initial total N (g)	Final Total N (g)	Total nitrogen loss (%)
CK	9350.3 ± 992.1	5808.3± 593.2	37.9 ± 1.1
T	8988.7±571.5	6404.0± 225.5	28.7 ± 2.2

Data indicate mean ± standard deviation based on three replications.

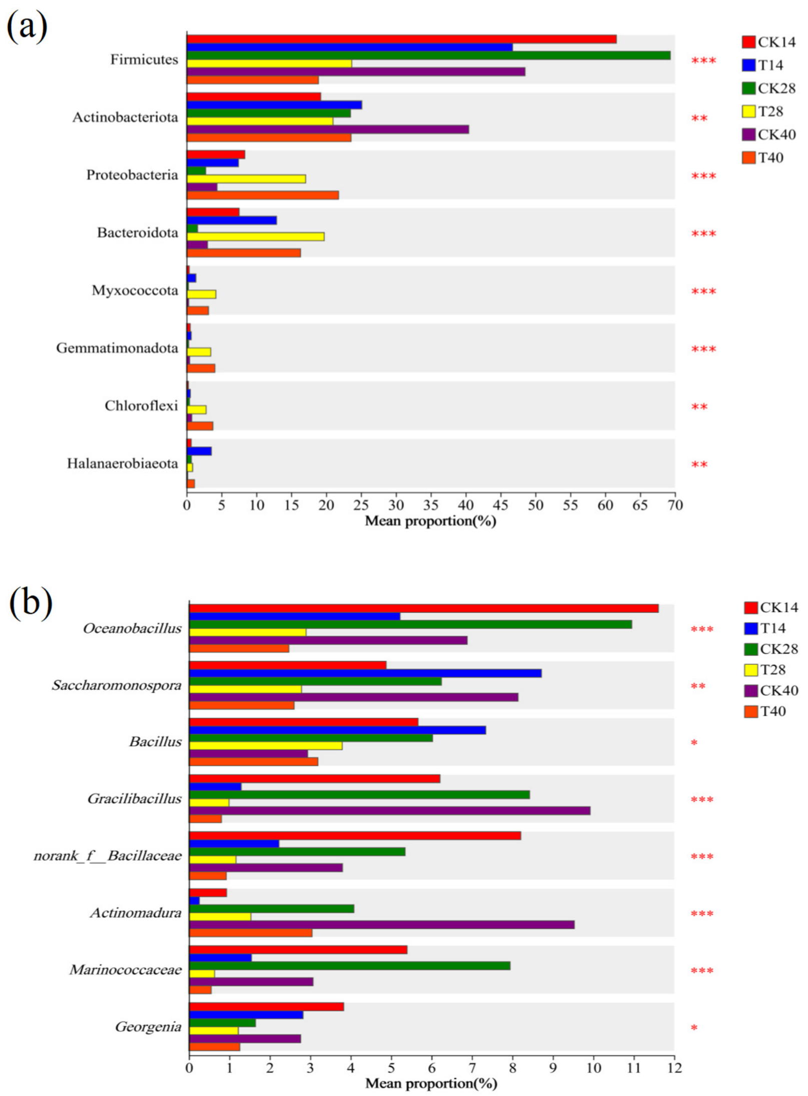

Figure S1. Significant differences in bacterial community composition at the (a) phylum and (b) genera levels when comparing the CK and T groups during composting, * P < 0.05, **P < 0.01, ***P < 0.001.
